# Results of the HCV self-testing implementation study through the secondary distribution of HCV self-tests among adult males in Georgia

**DOI:** 10.1186/s12889-026-26439-9

**Published:** 2026-04-01

**Authors:** Ketevan Stvilia, Rania A. Tohme, Sonjelle Shilton, Maia Tsereteli, Ketevan Galdavadze, Senad Handanagić, Irina Tskhomelidze Schumacher, Shaun Shadaker, Eter Kiguradze, Tamar Skhirtladze, Antons Mozalevskis, Niklas Luhmann

**Affiliations:** 1 National Center for Disease Control and Public Health of Georgia, Tbilisi, Georgia; 2 Division of Viral Hepatitis, Centers for Disease Control and Prevention, Atlanta, GA USA; 3 Operational and Implementation Research Unit, FIND, Geneva, Switzerland; 4 European Union Drugs Agency (EUDA), Lisbon, Portugal; 5 The Task Force for Global Health, United States, Tbilisi Office, Georgia; 6 Cancer Screening Center, Tbilisi, Georgia; 7 Testing, Prevention and Populations Team, Global HIV, Hepatitis and Sexually Transmitted Infections Programmes, World Health Organization, Geneva, Switzerland; 8 Agence Regionale Ile-de-France, CERGY, ILE DE FRANCE, France

**Keywords:** Acceptability, Hepatitis C, HCV, Self-testing, Testing strategy, Preferences

## Abstract

**Background:**

Scaling up hepatitis C virus (HCV) testing and diagnosis is a critical goal of the Global WHO elimination strategy. HCV self-testing (HCVST) has emerged as a promising self-care approach to improve access and early detection, particularly among hard-to-reach populations. In Georgia, a 2015 HCV serosurvey indicated that 57.3% of anti-HCV reactive individuals were men aged 30–59. This study evaluated a secondary distribution strategy for HCVST aimed at increasing testing uptake among this population in Tbilisi, Georgia.

**Methods:**

We implemented an observational cross-sectional study evaluating a secondary distribution delivery model of HCVST. We recruited women attending three cancer screening centers. Women who reported having a male family member or partner aged 30–59 years who had not previously been tested for HCV were provided with 1–3 oral fluid HCVST kits to distribute to these men, who were encouraged to complete the test, upload results to an online platform, and complete a questionnaire capturing demographic and risk-related data. Univariate and bivariate analyses were conducted to assess factors associated with reactive HCVST results. Linkage to care was evaluated via the national hepatitis C elimination program database six months after testing.

**Results:**

Between June 2022 and March 2023, 1,859 women were enrolled, distributing 2,744 kits. A total of 852 men uploaded test results; 723 (84.9%) completed the questionnaire. Of these, 16 (1.9%) reported a reactive result, 807 (94.7%) were non-reactive, and 29 (3.3%) indeterminate. Prior HCV testing was reported by 10.3% (*n* = 88). Reactive results were significantly associated with a history of imprisonment (*p* < .01) and injection drug use (*p* = .02). Of the 16 with reactive results, 10 were tested to confirm results at medical facilities, and 9 cases confirmed positive for anti-HCV. Seven (78%) were RNA-positive and initiated treatment.

**Conclusion:**

Secondary distribution of HCVST kits through women effectively engaged previously untested men and demonstrated a high rate of follow-up and treatment initiation. This strategy offers a promising model for expanding HCV testing coverage in Georgia. However, strengthened linkage-to-care mechanisms are essential to ensure timely confirmation and treatment of HCV infections identified through self-testing.

**Supplementary Information:**

The online version contains supplementary material available at 10.1186/s12889-026-26439-9.

## Background

The World Health Organization (WHO) estimates that 50 million people worldwide were living with chronic hepatitis C virus (HCV) infection, which is active infection and is characterized by the presence of both antibodies and HCV-RNA [1]. Globally, an estimated 36% of patients with chronic HCV infection have been diagnosed and 20% have been treated with direct-acting antivirals (DAAs) [1]. With regards to the WHO European Region,, an estimated 9 million people were living with chronic HCV infection, in 2022, however, only 29% had been diagnosed and 9% had been treated [1]. The WHO Global Health Sector Strategies on HIV, viral hepatitis, and sexually transmitted infections for the period 2022–2030 (GHSS) aim to eliminate hepatitis B and hepatitis C as public health threats by 2030 [2]. One of the key objectives of the Global WHO elimination strategy is to scale up the testing and diagnosis of HCV infection so that 90% of people living with chronic HCV infection know their status and that 80% of them are enrolled in treatment. Additionally, the strategy highlights the importance of health equity and reaching individuals most vulnerable to infection.

In Georgia, a nationally representative serosurvey conducted in 2015 estimated that the prevalence of chronic HCV infection was 5.4% among the adult population, and that 57.3% of antibody-to-HCV (anti-HCV) reactive persons in Georgia were men aged 30–59 years [3–5]. To mitigate this burden, Georgia launched a National Hepatitis C Elimination Program which provides all citizens with free testing and treatment with DAAs [6]. Georgia has also been proactively working to increase access to testing through decentralization and simplification of hepatitis C care pathways to facilitate linkage to hepatitis C care and treatment. By the end of December 2023, the program had made significant progress by screening 2.4 million adults (86% of the adult population). Based on an estimate 725,300 men aged 30–59 years in Georgia, 575,700 (79%) had been screened for anti-HCV. Hence, a remaining 21% of men in this age group are estimated to require screening for HCV infection. Barriers to testing uptake in Georgia include, a lack of awareness, widespread stigma, mistrust of the healthcare system, asymptomatic conditions, fear of being diagnosed with HCV infection, and fear of adverse health effects of treatment [7, 8]. Despite the wide availability of free access to HCV testing and treatment, additional innovative strategies are needed to address these barriers and improve access to HCV testing for certain target populations, including men aged 30–59 years.

Since 2021, the WHO has recommendsthe use of HCV self-testing (HCVST) as an additional approach to HCV testing services that may accelerate national and global progress toward elimination targets as it provides a mechanism to increase access to HCV testing, especially for those who have access challenges or feel mistrust around testing within health facilities [9]. HCVST is a process in which an individual collects their own samples (blood or oral fluid), performs a simple rapid diagnostic test (RDT), and then interprets the result. Self-testing often takes place in a private setting, either alone or with someone the individual trusts. HCVST is an antibody test; all reactive results must be followed with a second, repeated test for HCV antibodies and, if reactive, followed by a molecular test for HCV RNA by a trained healthcare provider according to the national algorithm for HCV infection diagnosis and treatment [10].

Although HIV self-testing (HIVST) has proven to be successful in improving HIV status awareness [11], not much is known about HCVST in the general population, or about successful distribution models for HCVST. A systematic review of HCVST studies has provided foundational evidence of the feasibility and acceptability of HCVST among populations disproportionally impacted by HCV infection in several countries [12]. In 2020, the National Center for Disease Control and Public Health of Georgia (NCDC) and the Foundation for Innovative New Diagnostics (FIND) completed two HCVST studies among persons who inject drugs (PWID) and men who have sex with men (MSM), which showed high feasibility, acceptability, and usability for both oral fluid and capillary specimen HCVST among both groups in Georgia [13]. In addition, a study conducted by FIND on values and preferences for HCVST across 10 low- and middle-income countries (LMICs) with communities most affected by HCV infection revealed that the benefits and advantages of HCVST far outweigh the potential disadvantages [14]. Most significantly, more than 94% of these study participants in five of the six countries (including Georgia) would recommend HCVST to friends and family [15].

Despite good evidence of the feasibility and acceptability of HCVST, implementation studies are needed to assess the effectiveness of some innovative HCVST distribution models in increasing access to HCV testing in general population groups not routinely engaged in healthcare.

Building on the evidence of studies demonstrating the feasibility of the secondary distribution of HIVST and in line with the recent WHO HCVST recommendations on program implementation [9], we conducted a study to assess the uptake of HCVST via a secondary distribution model.

## Methods

We implemented an observational cross-sectional study evaluating a secondary distribution delivery model of HCVST to evaluate a secondary distribution model of HCVST to evaluate HCVST uptake and operational considerations, HCV knowledge and risks, and linkage-to-care among men aged 30–59 years in Tbilisi Georgia by distributing HCVST to their female family members and partners.

We approached women attending three cancer screening sites in the capital city of Tbilisi for secondary distribution of HCVST to reach their male family members or partners. Cancer screening sites provide free onsite HCV testing to all individuals including women recruited for the study. The cancer clinics were selected on the basis of their location, size of catchment area, and size of the population they served. From June 2022 to the end of March 2023, 13,500 women were estimated to be attending the selected cancer screening sites. According to the number of HCVSTs provided for distribution within the study, and the test expiration date, the maximum number of women who could be recruited was 3,500. With this sample size, the study was designed to detect HCVST uptake and linkage to care with a statistical power of 95% at an alpha level of 0.05. The anticipated refusal rate of men who would receive the HCVST kit and would not upload their results or complete the online questionnaire was 30% based on previous local HCV studies [16, 17]. A single-stage purposive non-random sampling technique was utilized for the selection of women as primary participants. All women attending the selected cancer screening sites were asked HCV screening questions about the men they knew closely to establish eligibility for HCVST receipt. Women who were determined to be eligible based on the screening questions were recruited, provided informed consent for participation, and given at least one HCVST kit (ranging from 1–3 per woman based on choice) with printed instructions on how to use the test for distribution to eligible men. Participating women then distributed the HCVST kits to male family members and/or partners aged 30‒59 years.

### Eligibility criteria for female and male participants

Women aged 18 years or older were asked to participate if they were willing to recruit men who were partners/family members aged 30–59 years into the HCVST study and provided informed consent for study participation. During the piloting of the study protocol, we observed that women who tested positive for any type of cancer were less likely to agree to participate in the study because of the stress of their positive cancer screening; hence, a negative cancer screening result was used as eligibility for participation for women throughout the duration of the study.

The eligibility criteria for men as study participants were established through the selected female participants and it included the following: age 30–59 years; never having an anti-HCV test performed before the study; being eligible for the National Hepatitis C Elimination Program (having a valid personal ID or residence permit); having internet access to complete the online questionnaire and upload results; and able and willing to provide online informed consent.

### HCV self-testing

For this study, the rapid Antibody Test OraQuick® HCV Self-Test (Research-Use-Only)was used (the test received WHO prequalification for self-test use in July 2024). The OraQuick® HCV Rapid Test is a 20-min immunoassay for the qualitative detection of HCV antibodies in oral fluid [10]. Eligible male study participants who gave consent via the online platform were asked to collect his oral fluid samples and perform the HCVST. The HCVST package included illustrated instructions with simple, nontechnical language and instructions on how to register online to the study platform. All instructions were provided in Georgian language. Participants who reported reactive or indeterminate HCVST results were reached by the study team for counselling through a telephone call or online chat and were asked for detailed feedback on the test performance and reading of the results. They were then given recommendations to take a follow-up HCV rapid diagnostic test (RDT) performed by a trained provider at suggested medical facilities to confirm the results according to the national HCV testing algorithm of Georgia. After participants entered their HCVST result, HCV risk factor education automatically appeared on the online platform screen to educate them about HCV infection.

### Data collection and analysis

**Fig. 1:**
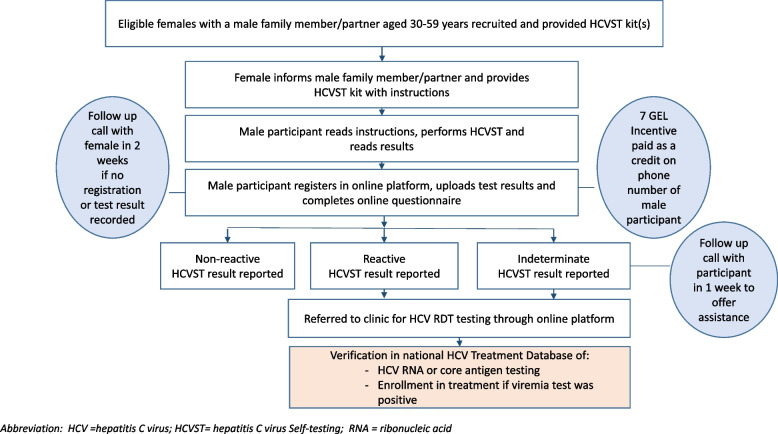
Flow diagram of participant enrollment for HCV self-testing in cancer screening centers and follow-up care, Tbilisi, Georgia, 2022–2023. Abbreviation: HCV = hepatitis C virus; HCVST = hepatitis C virus Self-testing; RNA = ribonucleic acid

Men who received HCVST kits could access the electronic study platform using the HCVST kit number and register with their personal ID and phone number. Data on male participants’ demographic information, knowledge about HCV infection, including risk factors for contracting HCV, and information on their past and current risk behaviors were collected via a structured online questionnaire integrated into the study web platform. The study participants were asked to report their test results. In addition, after completing an online questionnaire to share their experiences using the HCVST kit, they were given a small monetary incentive (7 GEL, or < 3 USD) in the form of credit on the phone account reported during registration on the study platform (Fig. 1).

The secure online platform with restricted access for only the study team provided additional built-in guidance for the study steps and follow-up steps for participants with positive HCVST results as well as the HCV risk related education information. Registration with personal ID allowed tracking of the participants’ progress along the HCV care continuum through the Hepatitis C Elimination Program Database (ELIMC) to check if they were previously tested for anti-HCV at a medical facility; if the participant registered on the platform but had not uploaded the result, the study team contacted him within two weeks and requested reporting of the result. If, after telephone communication, the result was not uploaded, it was regarded as a refusal. Within one week, a follow-up call was made with participants who uploaded an indeterminate test result to try to determine the reasons for not being able to read the result. HCV risk factor education section was automatically appearing on the online platform screen after reporting HCVST results.

Three months after enrollment of the last study participant, all participants who reported HCVST reactive results were followed up through the ELIMC to assess whether they initiated next steps to obtain a diagnosis and subsequent linkage to care and treatment for HCV infection if indicated. ELIMC is a national database that collects full information on hepatitis C diagnostic and treatment procedures in the country. The study participants’ data were fully protected on the online HCVST platform and in the ELIMC database.

### Statistical analysis

Descriptive statistics were used to summarize the characteristics of the study population and the HCVST results. Differences between the HCVST reactive and HCVST nonreactive study populations were compared with the chi-square test or Fisher's exact test for categorical variables and T-test for continued variables. All statistical tests were two-sided with an alpha of 0.05. The data were analyzed with IBM SPSS version 22. All study data were deidentified before analysis.

The main outcome of interest was the “result return rate,” or the proportion of distributed kits whose results were reported to the study team. We then characterized the remainder of the hepatitis C care cascade; we determined the proportion of returned HCVST results that were reactive, and the proportion of persons with reactive HCVST results who were subsequently linked to care for HCV RNA testing and initiation of DAA treatment.

In addition, we analyzed the participants’ views, concerns, and preferences regarding the use of HCVST as well as their history of risk behavior through an online questionnaire.

## Results

From June 2022 to March 2023, a total of 2,354 eligible women were asked to participate in the study and 1859 (78.9%) agreed. In total, 2,744 HCVST kits were distributed to those female participants, with an average of 1.5 kits (range 1–3) per female participant. Overall, 852 (45.8%) male family members or partners of the 1859 recruited women were registered on the electronic study platform and reported HCVST results. The recruitment of study participants varied by center; Center 1 and Center 3 each recruited more than 39% of all women and more than 36% of men. Center 2 was closed for approximately four months for renovation during the enrollment period, which resulted in a smaller number of recruited individuals (Table 1).

**Table 1:** Recruitment of participants and number of HCV self-tests (HCVSTs) distributed at study sites, Tbilisi, Georgia, 2022–2023

Cancer screening centers	Number & % of women recruited *N* = 1859		Number & % of HCVSTs distributed *N* = 2744		Number & % of men enrolled *N* = 852	
	N	%	N	%	N	%
Center 1	728	39.1	1044	38.0	313	36.7
Center 2	393	21.2	559	20.4	145	17.0
Center 3	738	39.7	1141	41.6	394	46.2

Among the 852 men who provided consent and were included in the study, 16 (1.9%) reported positive anti-HCV self-test results, 807 (94.7%) reported a nonreactive anti-HCV self-test results, and 29 (3.3%) could not could not read the test results due to blurry lines and reported indeterminate results. Among the 852 participants who reported test results, 10.3% (*n* = 88) reported having been tested for HCV infection before the study and were excluded from further analysis; thus, the total number of participants included in the analysis was 764. All 16 HCVST-reactive individuals reported either not being tested previously (87.5%, n = 14) or not being aware if they were previously tested for HCV infection (12.5%, *n* = 2).^1^

Among the 764 men who uploaded their HCVST results and were not tested for HCV infection previously, 94.6% (n = 723) completed the online questionnaire. The median age of the male participants was 44 years (IQR 14), 96.2% were Georgian, 89.0% were employed, and 60.2% had a college degree. The sociodemographic characteristics of the HCVST-reactive participants did not differ significantly from those of the HCVST-nonreactive participants (Table 2). Among the 717 participants who described their previous testing experience, 70.3% (*n* = 508) reported that they were never offered testing for HCV before this study, 17.7% (*n* = 128) said that they did not remember if they were offered testing, and 11.2% (*n* = 81) reported that they previously refused to be tested for HCV. The main reasons for not having previously tested for HCV were not considering themselves to be at risk (53.6%, *n* = 391), not being interested in tested (26.7%, *n* = 194), or not having time to go to a medical facility for testing (13.2%, *n* = 96).

**Table 2:** Sociodemographic characteristics and previous HCV testing experience among men recruited for HCV self-testing who responded to the online questionnaire, Tbilisi, Georgia, 2022–2023

Sociodemographic characteristics	Total *N* = 723*		HCVST non-reactive Participants, *N* = 678		HCVST reactive Participants, *N* = 16		*P* value
	**n**	**%**	**n**	**%**	**n**	**%**	
Median Age (IQR)	**44 (IQR 14)**		**44 (IQR 13)**		**46 (IQR 6)**		
Age of male participants, years							
< 30	9	1.2	9	1.3	0	0.0	
30–39	227	31.4	208	30.7	5	31.3	
40–49	265	36.7	254	37.5	5	31.3	.53
50–59	210	29.0	195	28.8	6	37.5	
> 59	12	1.7	12	1.8	0	0.0	
Ethnicity							
Georgian	696	96.2	651	96.0	16	100.0	.50
Other	27	3.7	27	4.0	0	0.0	
Employment Status							
Employed	412	57.0	383	56.5	10	62.5	
Self-employed	231	32.0	219	32.3	5	31.3	
Unemployed	72	10.0	68	10.0	1	6.3	.48
Pensioner	6	0.8	6	0.9	0	0.0	
Other	2	0.3	2	0.3	0	0.0	
Level of Education							
No school education	0	0.0	0	0	0	0.0	
Less than high school (grades 1–10)	37	5.1	37	5.5	0	0.0	
High school (grades 11–12)	127	17.6	117	17.3	3	18.8	.70
Professional/technical school	115	15.9	109	16.1	3	18.8	
University education (completed)	435	60.2	406	60.0	10	62.5	
Refused to answer	9	1.2	9	1.3	0	0.0	
Were you offered HCV testing sometime before?							
No, never	508	70.3	486	71.7	0	0.0	
Yes, but I didn’t want to test	81	11.2	76	11.2	9	56.3	.04
Others (specify)	6	0.8	6	0.9	0	0.0	
Don’t know	128	17.7	110	16.2	7	43.8	
If not tested before for HCV, why? (Multiple responses)							
Do not see myself at risk	391	53.6	376	55.5	4	25.0	
Have not been interested	194	26.7	185	27.3	6	37.5	
Do not have time to go to a testing center	96	13.2	85	12.5	3	18.8	.26
Do not know how to get tested	24	3.3	24	3.5	0	0.0	
Afraid of testing HCV positive	14	1.9	12	1.8	0	0.0	
Afraid of stigma and/or discrimination	8	1.1	7	1.0	1	6.3	
Other	7	1.0	3	0.4	2	12.5	
Don’t know	115	15.8	104	15.3	0	0.0	

*Abbreviations*: *HCV* hepatitis C virus, *HCVST* HCV self-testing, *IQR* interquartile range

^*^Total number 723 includes participants who reported reactive, nonreactive and indeterminate HCV self-test results

Among individuals with HCVST reactive results, 6 (37.5%) reported not being previously tested due to a lack of interest in HCV testing, 4 (25.0%) did not consider themselves at risk of HCV infection, and 3 (18.8%) said that they did not have time to get tested at a clinic. A greater percentage of participants who reported HCVST-reactive results had been offered HCV testing prior to this study but refused it, compared to those who were HCVST-nonreactive (56.3% vs 11.2%, *p* = 0.04).

### Usability and acceptability of HCVST

Among the 723 men who completed the online questionnaire, 703 (97.2%) provided feedback on their HCVST experience. More than half (58.9%; *n* = 414) performed the HCVST independently, 38.5% (*n* = 271) required a family member's help and 1.8% (*n* = 13) received help from the study team. The majority (97.7%, *n* = 687) understood their test results.

**Fig. 2:**
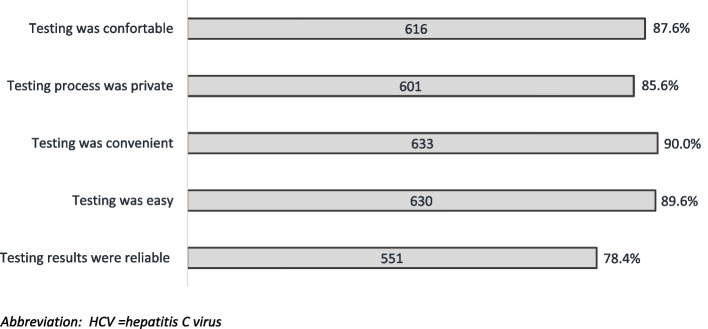
Feedback of study participants on the HCV self-testing process, Tbilisi, Georgia, 2022–2023 (*n* = 703). Abbreviation: HCV = hepatitis C virus

Among 703 participants who shared feedback on the HCVST procedure, 87.6% (*n* = 616) reported that testing was either comfortable or very comfortable, 85.6% (*n* = 602) enjoyed the privacy of testing at home, 90.0% (*n* = 633) found it convenient to have the possibility of performing the test when they wanted, 89.6% (*n* = 630) reported that testing was either easy or very easy, and 74.2% (*n* = 551) believed that the HCVST result was reliable (Fig. 2).

Furthermore, 89.9% (*n* = 632) reported that they would use another HCVST kit if it was provided; (Table 3).

**Table 3:** Preferences and desired frequency of HCV self-testing among recruited men in Tbilisi, Georgia, 2022–2023

	Whole sample		HCVST nonreactive participants		HCVST reactive participants		*P* value
Would you test yourself at home for HCV if you had a testing kit and instructions on how to do it?	*N* = 03	%	*N* = 674	%	*N* = 16	%	
Yes	632	89.9	609	90.4	14	87.5	.95
No	12	1.7	9	1.3	1	6.3	
Don’t know	63	9.8	56	8.3	1	6.3	
Reported preferred place for getting tested with HCVST	*N* = 632	%	*N* = 609	%	*N* = 14	%	.70
At home without assistance	210	33.2	208	34.2	4	28.6	
At home with someone they trust	185	29.3	180	29.6	3	21.4	
In a health care clinic by trained HCW	80	12.7	77	12.6	2	14.3	
No preference	135	21.4	133	21.8	2	14.3	
Other	22	3.4	11	1.8	3	21.4	

*Abbreviations*: *HCVST* hepatitis C virus self-testing, *HCW* healthcare worker

When asked about the preferred place for self-testing, the majority (62.5%; *n* = 395) reported a desire to get tested at home, including 185 (29.3%) individuals who would like to receive assistance from somebody they trust. Only 12.7% (*n* = 80) said that they would prefer HCVST to be performed by a healthcare professional at a medical facility, whereas 21.4%, (*n* = 135) did not report any preference for a testing place (Table 3).

Among the 29 individuals who could not interpret their test results, 19 completed the online questionnaire. Among them, 13 (68.4%) would still be retested with HCVST if the kit was provided.

Among the 13 participants who completed the questionnaire but did not perform the test, three (23.1%) cited a lack of time, three (23.1%) did not know how to use the test, two (15.4%) were not interested, one (7.7%) was afraid of being tested, and four (30.8%) participants did not specify a reason for not using the provided HCVST kit.

### Risk factors

A total of 643 participants responded to the risk factors questions. Among the group of individuals with HCVST-reactive results, one reported previous injection drug use and all reported receiving dental care and injections for medical purposes (Table 4).

**Table 4:** Reported risk factors for HCV infection among study participants, 2022–2023

HCV risk factor	Total		HCVST nonreactive participants		HCVST reactive participants		*P* value
Have you ever injected not prescribed drugs for nonmedical purposes?	*N* = 643	%	*N* = 609	%	*N* = 14	%	.02*
Yes	7	1.1	5	0.8	1	7.1	
How many sexual partners did you have during the last 6 months? (mean, range)	1.06 (0–10)		1.05 (0–10)		1.21(1–4)		.36
How often did you use condoms with your sexual partner(s)?	N = 610	%	N = 578	%	N = 16	%	
Always	148	24.3	135	23.4	4	25.0	.55
Rarely	301	49.3	291	50.3	5	31.2	
Never	161	26.4	152	26.3	7	43.8	
Have you ever been transfused a blood or blood products?	N = 609	%	N = 578	%	N = 16	%	
Yes	37	6.1	33	5.7	2	12.5	.23
During the last 6 months did you shave in a salon/barber shop?	N = 639	%	N = 606	%	N = 16	%	
Yes	62	9,7	57	9.4	1	6.3	.77
Have you ever had a surgery?	N = 67	%	N = 62	%	N = 16	%	
Yes	51	76.1	48	77.4	1	6.3	.31
Did you ever receive dental care?	N = 643	%	N = 610	%	N = 16	%	
Yes	587	91.3	558	91.5	15	93.8	.85
Did you ever inject drugs for medical purposes?	N = 643	%	N = 610	%	N = 16	%	
Yes	561	87.2	532	87.2	14	87.5	.87
Have you ever been imprisoned?	N = 637	%	N = 604	%	N = 16	%	<.01*
Yes	25	3.9	21	3.5	4	25.0	
Do you have a tattoo?	N = 637	%	N = 604	%	N = 16	%	.24
Yes	73	11.5	68	11.3	3	18.8	
Did you share any of the following items with a family member? (multiple response)	N = 637	%	N = 604	%	N = 16	%	
Toothbrush, Yes	21	3.3	18	3.0	1	6.3	.05*
Towel, Yes	301	47.3	288	47.7	5	31.2	.97
Shaving kit, Yes	48	7.5	44	7.3	3	18.8	.05
Scissors, Yes	260	40.8	246	40.7	4	25.0	.92
Nail kit, Yes	228	35.6	217	35.9	4	25.0	.95

The associated *p* value was <.05, suggesting a statistically significant association with HCVST reactivity

Compared with participants who were HCVST nonreactive, a greater percentage of those who were HCVST reactive reported ever injecting drugs (7.1% vs. 0.8%, *p* = 0.02), ever being incarcerated (25.0% vs. 3.5%, *p* < 0.01), and sharing a toothbrush (6.3% vs. 3.0%, *p* = 0.05).

### Linkage to medical facility-based testing, diagnosis and treatment

Within six months after completing their HCVST, all 16 individuals whose HCVST results were positive were followed up in the ELIMC database to assess their follow-up for HCV diagnosis and treatment. Overall, 10 (62.5%) individuals went to a medical facility to receive a professional anti-HCV test, of whom 9 (90.0%) were reactive. HCV RNA testing was performed for all 9 patients (100.0%), of which 7 (77.8%) were confirmed to have current HCV infection and were started on DAA treatment. 3

## Discussion

This study provides robust evidence supporting the implementation potential of hepatitis C self-testing (HCVST) as an innovative and scalable public health strategy. By targeting male partners of women attending health facilities—an often-overlooked group in conventional healthcare delivery—the study successfully reached a population that has historically demonstrated lower levels of engagement with facility-based HCV testing. Among participants who completed the online questionnaire, 70.5% reported never having been offered an HCV test, and an additional 17.6% could not recall ever being offered testing. These findings underscore persistent gaps in awareness and coverage, even after more than eight years of Georgia's national hepatitis C elimination program. They also highlight the limitations of conventional testing strategies in reaching disengaged populations and position HCVST as an important potential tool for identifying undiagnosed infections in underserved groups.

Participants’ feedback further affirmed the acceptability and user-friendliness of the HCVST intervention. More than 85% of those who completed the study questionnaire described the self-testing process as easy to use, comfortable, private, and convenient—factors especially important for men, who may face stigma and privacy concerns related to HCV. Furthermore, 78% expressed confidence in the reliability of their results, and 89.6% stated they would use a self-test again if provided with a kit. This strong endorsement supports the integration of HCVST into national screening strategies and reinforces its potential to increase both initial and repeat engagement in testing programs.

A notable innovation in this study was the exploration of secondary distribution, whereby HCVST kits were distributed to male partners or family members via female participants attending health facilities. Nearly 80% of women at cancer screening sites in Tbilisi accepted kits for secondary distribution. However, only 45.8% of the intended recipients reported their results through the electronic platform, though 16 of these reported reactive results.

Importantly, the study demonstrated better outcomes in linkage to care following self-testing than number of similar studies conducted in other settings [18, 19]. Among participants who reported a reactive HCVST result, 63% proceeded to confirmatory testing at a healthcare facility—a level of follow-up that is encouraging given the decentralized and self-directed nature of the intervention. Of those who sought confirmatory testing, 90% were confirmed anti-HCV reactive, and all individuals diagnosed with chronic infection successfully initiated treatment. These outcomes are broadly comparable to Georgia’s national hepatitis C elimination program, where 87.6% of anti-HCV reactive individuals undergo RNA or core antigen testing and 85.7% of those with confirmed infection start treatment. These results highlight the potential of HCVST, even in non-traditional settings, to achieve effective linkage to care and support treatment initiation at levels similar to established facility-based programs.

Despite these encouraging parallels, the gap between self-testing and clinical confirmation underscores the need for integrated support systems within HCVST programs. Digital navigation tools, SMS reminders, community outreach, and peer-led models could all help bridge this gap and improve continuity of care.

While secondary distribution has been extensively evaluated in HIV self-testing (HIVST) programs—especially in antenatal settings [20–23], this study is among the first to assess its feasibility for HCV. The observed result reporting rate indicates the importance of coupling distribution with proactive follow-up and support strategies, such as those used in HIVST programs, including financial incentives and structured result collection mechanisms.

The study’s risk factor analysis was consistent with findings from national surveys conducted in 2015 and 2021, which showed that anti-HCV reactivity was significantly associated with a history of injection drug use and incarceration [6, 24]. These associations reaffirm the importance of prioritizing high-risk populations—such as people who inject drugs, currently or formerly incarcerated individuals, and other marginalized groups including migrants, adolescents, and men who have sex with men—in future HCVST expansion efforts. Tailored outreach and targeted distribution strategies in these communities could yield the highest return in identifying undiagnosed cases.

From both clinical and policy perspectives, these findings carry important implications. Georgia’s national serosurvey conducted in 2021 documented a substantial reduction in HCV RNA prevalence among men—from 9.0% in 2015 to 3.1%—marking a major public health achievement [6]. However, this success also highlights the growing challenge of locating the remaining undiagnosed individuals, many of whom likely belong to hidden or underserved populations. In this context, the findings of the current study are highly relevant: they demonstrate that HCVST—particularly when coupled with secondary distribution—offers a promising alternative approach to reach individuals who have remained outside the reach of conventional facility-based testing. This strategy could play a pivotal role in sustaining progress toward elimination and addressing the final gaps in diagnosis and care.

Moreover, the study’s outcomes are consistent with previous research showing the effectiveness of involving family members in engaging hard-to-reach individuals in HCV care. Compared to traditional facility-based testing strategies, the secondary distribution model in this study achieved similar rates of confirmatory testing and treatment initiation among HCVST-reactive individuals. These findings further validate the potential of family-centered approaches in expanding hepatitis C testing coverage.

Future research should evaluate the cost-effectiveness, long-term health outcomes, and scalability of HCVST within diverse health systems. Expanding this approach to additional underserved or high-risk groups will be critical to maximizing public health impact. As countries strive to meet the WHO's hepatitis C elimination targets by 2030, the integration of decentralized, user-driven strategies such as HCVST—strengthened by strong linkage mechanisms and targeted outreach—could be transformative in identifying and treating the final pool of undiagnosed infections.

### Study limitations

The main disadvantage of the purposive sampling method is selection bias, as facility and participant recruitment relied on subjective decisions. To reduce this risk, we clearly defined eligibility criteria. The study depended on female family members’ knowledge of their male relatives’ HCV status, which may have limited male participation and led to the inclusion of some previously tested men—sometimes without their own or the women’s awareness of prior testing. Although men who reported prior HCV testing were excluded, the absence of linkage to the National HCV Program database prevented verification of testing history.

Nonresponse bias is also possible: men who did not use or report results, particularly reactive results, may have differed from those who participated, potentially experiencing unreported testing difficulties. As testing occurred in participants’ homes, all reported results were self-interpreted, consistent with HCVST’s aim to increase awareness by offering private testing to reduce stigma. However, fear of disclosing results to family members may have discouraged participation among men aware of their higher HCV risk.

The requirement for online result reporting may have excluded individuals less comfortable with digital platforms. The concentration of cancer screening sites in the capital city may have reduced participation from residents in outlying areas and other regions. Finally, language barriers were noted among some Azeri women who could not speak, read, or understand Georgian, affecting engagement at screening sites.

## Conclusion

In summary, hepatitis C self-testing presents a promising strategy to expand access to diagnosis and treatment, particularly among populations not well served by traditional healthcare systems. This study highlights its potential to identify previously undiagnosed infections, overcome testing barriers, and align with harm reduction efforts. However, successful scale-up will require deliberate planning to ensure strong linkage to confirmatory testing and care, along with integration with HIVST or into broader health systems and public health strategies. As countries consider incorporating HCVST into national programs, ongoing evaluation, targeted outreach, and adaptive implementation will be key to maximizing its impact and advancing hepatitis C elimination goals.

## Supplementary Information

Below is the link to the electronic supplementary material.

Supplementary Material 1

## Data Availability

The data can be requested from corresponding author. Data with personally identifiable information cannot be shared.

## Abbreviations

Anti-HCV: Antibody to hepatitis C virus
DAA: Direct-acting antiviral medications
ELIMC: National Hepatitis C Elimination Program Database
FIND: Foundation for Innovative New Diagnostics
HCV: Hepatitis C virus
HCVST: Hepatitis C virus self-testing
LMIC: Low- and middle-income countries
RNA: Ribonucleic acid
WHO: World Health Organization

## Footnotes

1 Hospital patients are tested on multiple infections and the test results are provided only if it is reactive, they maybe not aware/don’t remember if they were tested before for HCV
