# Correction: Results of the HCV self-testing implementation study through the secondary distribution of HCV self-tests among adult males in Georgia

**DOI:** 10.1186/s12889-026-27971-4

**Published:** 2026-07-16

**Authors:** Ketevan Stvilia, Rania A. Tohme, Sonjelle Shilton, Maia Tsereteli, Ketevan Galdavadze, Senad Handanagić, Irina Tskhomelidze Schumacher, Shaun Shadaker, Eter Kiguradze, Tamar Skhirtladze, Antons Mozalevskis, Niklas Luhmann

**Affiliations:** 1 National Center for Disease Control and Public Health of Georgia, Tbilisi, Georgia; 2 Division of Viral Hepatitis, Centers for Disease Control and Prevention, Atlanta, GA USA; 3 Operational and Implementation Research Unit, FIND, Geneva, Switzerland; 4 European Union Drugs Agency (EUDA), Lisbon, Portugal; 5 The Task Force for Global Health, United States, Tbilisi Office, Georgia; 6 Cancer Screening Center, Tbilisi, Georgia; 7 Testing, Prevention and Populations Team, Global HIV, Hepatitis and Sexually Transmitted Infections Programmes, World Health Organization, Geneva, Switzerland; 8 Agence Regionale Ile-de-France, CERGY, ILE DE FRANCE France

**Correction: BMC Public Health 26**,** 1533 (2026)**

**https://doi.org/10.1186/s12889-026-26439-9**

Following publication of the original article [1], an error was reported in the copyright line and the licence subtype needs to be updated. The correct copyright line should be “© World Health Organization and the Authors 2026” and the correct licence subtype should be “CC BY 3.0 IGO”.

The original article [1] has been updated.
