# Supplementary material for: Results of the HCV self-testing implementation study through the secondary distribution of HCV self-tests among adult males in Georgia
Source: BMC Public Health. 2026 Apr 1;26:1533. doi: 10.1186/s12889-026-26439-9 (PMC13169970; doi:10.1186/s12889-026-26439-9)
Supplement: Supplementary file 1 — Supplementary Material 1 [file 12889_2026_26439_MOESM1_ESM.pdf]

Annex 1. The HCV self-testing implementation study through the secondary distribution of HCV self-tests among adult males in Georgia

Online Questionnaire

Section A	Socio demographic characteristics	
A1	**What is your age?**	
A2	**What is your nationality?**	
		1. Georgian
		2. Armenian
		3. Azari
		4. Russian
		5. Other (please specify)
		9. No response
A3	**What is your employment status?**	
		1. employed
		2. self-employed
		3. Unemployed
		4. Pensioner
		5. Other (please specify)
		9. No response
A4	**What is your marital status?**	1. Single
		2. Married
		3. Widowed
		4. Separated
		5. In a relationship
		9. No response
A5	**What is your education level?**	
		1. Secondary School (1-6 grades)
		2. uncomplete high school (7-10 grades)
		3. High school (11-12 grades)
		4. No school education
		5. Professional/technical school
		6. University education (completed)
		7. don't know/don't remember
		9. refused to answer

Section B	History of HCV testing				
B1	**Ever tested for HCV before the study**	1. Yes	2. No	3. Don't know	4. No response
B2	**If yes, what was the testing result**				
		1. Positive			
		2. Negative			
		3. Don't know/ don't remember			
		9. No response			

B3	**Were you offered HCV testing sometime before?**				
		1. No, never			
		2. Yes, but I didn't want to test			
		3. Others (specify)			
		4. Don't know			
		9. No response			
B4	**If not tested before for hepatitis C, Why? (all that apply)**				
		1. Do not see myself at risk			
		2. Do not know how to get tested			
		3. Have not been interested			
		4. Do not have time to go to a testing centre			
		5. Afraid of testing hepatitis C positive			
		6. Afraid of stigma and/or discrimination if I go to a testing centre and ask for a hepatitis C test			
		7. Other			
		8. Don't know			
		9. No response			
B5	**Where would you most prefer to be tested for hepatitis C?**				
		1. By myself at home			
		2. At home with someone I trust			
		3. By myself at a healthcare clinic			
		4. In a community centre by community-based organization staff			
		5. In a healthcare clinic by a healthcare worker			
		6. In a pharmacy by a healthcare worker			
		7. No preference			
		8. Prefer not to get tested for hepatitis C			
		9. Other			
		99. No response			
**Section C**	**HCV self-testing experience**				
C1	**Did you complete the hepatitis C testing that was offered to you as part of this study?**	1.Yes	2.No	3.Don't know	4.No response
C2	**If yes, the test result was**				
		1. Positive			
		2. Negative			
		3. Test did not work			
		4. Don't know, could not read the test			
		5. Do not want to disclose			
C3	**Did you understand the test result?**				
		1. Yes	2. No	3. Don't know	
C4	**If you didn't test, why not? (All that apply)**				
		1. Did not want to test/was not interested			
		2. Forgot to get tested			
		3. Afraid of testing			
		4. Did not have time			
		5. Didn't know how to use the provided test kit			

		6. Other (specify)
		9.No response
C5	**If you didn't use ST kit, did you try to get HCV test somewhere else?**	
		1. Yes, went for testing to the Cancer Screening site where my family member was provided with ST kit
		2. Yes, went for the specialized HCV treatment Clinic
		3. No, didn't do any steps for getting tested
		4. Other
		9.No response
C6	**Did you used somebodies help for performing HCV self-test?**	
		1. Yes, I contacted the study team (on phone)<br>2. Yes, I asked a friend/family member for help<br>3. Yes, I search for information online<br>4. Other (specify)<br>5. No, I performed self-testing independently<br>9.No response
C7	**How easy was the testing process?**	
		1 Not very easy
		2
		3 Average
		4
		5 Very easy
C8	**How convenient was the testing process?**	1 Not very convenient<br>2<br>3 Average<br>4<br>5 Very convenient
C9	**How private did you think the testing process was?**	
		1 Not very private
		2
		3 Average
		4
		5 Very private
C10	**How much do you feel you can trust the test results?**	
		1 Not very trustworthy
		2
		3 Average
		4
		5 Very trustworthy
C11	**How comfortable was the testing process?**	
		1 Not very comfortable
		2
		3 Average
		4
		5 Very comfortable
C12	**In the future, where would you prefer to be tested for hepatitis C?**	

		1. By myself at home		
		2. At home with someone I trust		
		3. By myself at a healthcare clinic		
		4. In a healthcare clinic by a healthcare worker		
		5. In a pharmacy by a healthcare worker		
		6. No preference<br>.		
		7. Prefer not to get tested for hepatitis C		
		8. Other (specify)		
		9. No response		
C13	**Would you test yourself at home for hepatitis C if you had a testing kit and instructions on how to do it?**			
	Yes	1. yes	2. No	3. Don't know
C14	**If yes, how often do you think you would test yourself?**			
		1. More than once every 6 months		
		2. Once every 6 months		
		3. Once a year		
		4. Once every 2 years		
		5. Don't know		
		6. Other (specify)		
**Section D**	**Risk Factors for Hepatitis C**			
D1	**Have you ever injected prescribed drugs for non-medical purposes?**	1. Yes	2. No	3. No Response
D2	**If yes, how often did you injected prescribed drugs?**			
		1. Once a month		
		2. Once a week		
		3. 2-3 times a week		
		4. 4-5 times a week		
		5. Once a day		
		6. Several times a day		
		7. Did not inject last month		
		8. Don't know		
		9. Do not inject drugs any more		
D3	**If yes, In the past 6 months, have you ever used a needle/syringe that was used by somebody else before?**			
	Yes	1. Yes	2. No	3. Don't know/don't remember
D4	**How many sexual partner did you have during the last 6 months**			
D5	**How often did you use condom with your sexual partner/s**	1. Always	2. Never	3. Rarely
D6	**Have you ever been transfused a blood or blood products**	1. Yes	2. No	3. Don't know/don't remember

D7	**During the last 6 months did you shaved in a salon/barbers shop**	1. Yes	2. No	3. Don’t know/don’t remember
D8	**Did stylist use new shaving instruments?**	1. Yes	2. No	3. Don’t know/don’t remember
D9	**Have you ever had a surgery**	1. Yes	2. No	3. Don’t know/don’t remember
D10	**Did you ever received a dental care**	1. Yes	2. No	3. Don’t know/don’t remember
D11	**Did you ever injected drug for medical purposes?**	1. Yes	2. No	3. Don’t know/don’t remember
D12	**Have you ever been imprisoned?**	1. Yes	2. No	
D13	**Do you have a tattoo?**	1. Yes	2. No	
D14	**Did you share any of the following items with a family member**			
	1. Tooth brush	1. Yes	2. No	3. Don’t know/don’t remember
	2. Towel	1. Yes	2. No	3. Don’t know/don’t remember
	3. Shaving kit	1. Yes	2. No	3. Don’t know/don’t remember
	4. Seizers	1. Yes	2. No	3. Don’t know/don’t remember
	5. Neil kit	1. Yes	2. No	3. .Don’t know/don’t remember
